# Hermeneutical injustice and unworlding in Psychopathology

**DOI:** 10.1080/09515089.2023.2166821

**Published:** 2023-01-21

**Authors:** Lucienne Jeannette Spencer

**Affiliations:** Institute of Mental Health, University of Birmingham, U.K

**Keywords:** Phenomenology, epistemic injustice, hermeneutical injustice, unworlding, philosophy of psychiatry, Merleau-Ponty

## Abstract

The rich literature in phenomenological psychopathology regards the communicative difficulties accompanying psychiatric illness as a product of ‘unworlding‘: the experience of a drastic change in one’s habitual field of experience. This paper argues that the relationship between speech expression and unworlding in psychiatric illness is more complex than previously assumed. Not only does unworlding cause a breakdown in speech expression, but a breakdown in speech expression can perpetuate, and even exacerbate, the experience of unworlding characteristic of psychiatric illness. In other words, I identify a two-way relationship between unworlding and the communication breakdown in psychiatric illness. Merleau-Ponty’s phenomenology of speech expression is drawn upon to demonstrate how hermeneutical injustice in psychiatric healthcare can elicit unworlding for the person with a psychiatric illness.

## Introduction

There is a long tradition of employing a phenomenological approach to gain greater insight into the unique experience of psychiatric illness. Researchers in this field have shed light upon a disturbance in the overall structure of experience characteristic of psychiatric illness, which causes the embodied subject to encounter the world in a fundamentally different way from their neuro-normative counterparts. This disturbance can be referred to as “unworlding”, a term popularized by Louis Sass (1990) but derived from Heidegger (1985, p. 196).

The paper aims to better understand the relationship between “unworlding” and ineffability in psychiatric illness. Drawing on Merleau-Ponty’s phenomenology of speech expression, I argue that “unworlding” not only causes a breakdown in speech expression but a breakdown in speech expression can perpetuate, and even exacerbate, the experience of “unworlding” characteristic of psychiatric illness. In other words, I identify a two-way relationship between “unworlding” and the communication breakdown in psychiatric illness.

This paper begins with a brief account of “unworlding” and its application in phenomenological psychopathology. It then illuminates the unequal hermeneutical landscape in psychiatric healthcare that drives inexpressibility. I identify this as hermeneutical injustice. The final two sections consider how hermeneutical injustice can constitute a breakdown in the body schema for the person with psychiatric illness due to the deprivation of an essential bodily capacity: speech expression. Thus, I demonstrate how hermeneutical injustice can give rise to “unworlding” in psychiatric illness.

## Unworlding

To understand how one can experience a disconnect between self a world, we first need to examine what it’s like to feel a self-world *synthesis*. The concept of “being-in-the-world” was first introduced by Heidegger to denote the cohesive whole of a subject and their environment. In addition, feeling at home in the world also consists of cohesion in one’s body, time and space. According to Heidegger, the subject (*Dasein*) does not merely “inhabit” the world, like a person positioned in a space, but “dwells” in the world, like a person belonging to a home and experiencing a “simple oneness” with their surroundings: “*ich bin, du bist* means: I dwell, you dwell. The way in which you are and I am, the manner in which we humans are on the earth, is *Bauen*, dwelling” (Heidegger, 1971, p. 147). For Heidegger, the world does not merely denote the environment *Dasein* finds herself in but the other people and objects that make up that world. To dwell is to experience a cohesive web of interrelations between *Dasein*, objects and other people that make up the world. In dwelling, other people and objects are experienced not only as useful things that may assist our existence but as things-in-themselves.

Building on the work of Heidegger, Merleau-Ponty posits that meaning structures immerse the *embodied* subject in the world and allow them to move through it with a pre-reflective openness. Consider Merleau-Ponty’s example of walking through the streets of Paris: “the cafes, the faces, the poplars along the quays, the bends of the Seine- it is cut out of the total being of Paris” (Merleau-Ponty, 2012, p. 294). For Merleau-Ponty, the cafes, the faces and the poplars make up a cohesive and meaningful whole that is Paris. The individual features become no more distinct to him than “the eyes of a familiar face”; these independent objects make up the totality of Paris for the Parisian (ibid). Merleau-Ponty’s embodied activity in Paris (sitting in its cafés, smelling its poplars, talking to its people) is supported by this web-of-meaning. The meaning structures of Paris are mediated by Merleau-Ponty’s embodied engagement with it. Merleau-Ponty experiences “the bends of the Seine” as it relates to the poplars, the surrounding buildings, and Parisiennes, even experiencing how the Seine relates to the landscape, the history and the identity of Paris itself. In this rich and dynamic engagement with the world, the embodied subject that is Merleau-Ponty pre-reflectively grasps all these meaning-structures all at once.

Yet, such a cohesive experience of worldhood is vulnerable to collapse into a state of “unworld”, thus disrupting the experience of dwelling. This “unworlding” captures transformative changes in the structure of *Dasein’s* lived world of space, time, objects, atmosphere, body, intersubjectivity and intentionality (how the world offers possibilities for action to *Dasein*). The example of unworlding that Heidegger provides is that of *Dasein’s* existence during an era of war: “the ‘world’ has become an unworld as a consequence of the abandonment of beings by Being’s truth” (Heidegger, 1993). Drawing on Heidegger, Sass fleshes out the concept of “unworlding”, whereby “external reality loses … its human resources or significance” (Sass, 2017, p. 16). While the two concepts may often overlap, Sass makes an important distinction between “unworlding” and “derealization”; in an instance of derealization, the world loses its “otherness”, or “its ontological status as an entity or horizon independent of the perceiving subject” (ibid). However, in a case of “unworlding”, it is the “aura of significance” that is lost (ibid).^1^ Turning back to Merleau-Ponty’s example of walking through the streets of Paris, one can imagine, in a state of unworlding, the familiar becomes alien: people’s faces seem strange and inscrutable; the bends of the Seine feel invasive of one’s space; the poplars, cafes and other Parisienne objects no longer feel part of a cohesive environment but distinct and detached etc.

Although various terms have been employed in the literature, this experience of the subject being unrooted from the world has been explored in multiple contexts, such as the female experience (Young, 2005) and the Black experience (Fanon, 1952). However, the application that has gained the most attention is in the field of psychiatric illness. In what follows, I will provide a comprehensive overview of the current work on “unwordling” as it occurs in a psychiatric illness.

## Unworlding in psychiatric illness

As established in the work of Havi Carel, the “unworlding” that arises from the disruption of bodily experience is most apparent in cases of somatic illness (Carel, 2016). Illness obstructs a kind of bodily certainty that would normally allow a non-disabled person to interact with the world with a certain confidence in one’s corporal abilities. Instead, in illness, the person is pervaded with a “bodily doubt” that throws into question one’s bodily capacity to accomplish the task at hand (Carel, 2016, p. 87). As such, the freedom to engage with the world is compromised as specific bodily actions are hampered. The ill subject finds themselves cut off from certain affordances offered by the world. For the person who has lost the ability to walk, stairs no longer invite the possibility of being climbed. For the person who experiences breathlessness, a steady slope may present itself as an arduous trek (Carel, 2016, p. 76). Somatic illness roots the person to their body as habitual acts, such as crossing the room, now require a reflective, corporal determination to accomplish the action. The ill-body can no longer enjoy the transparency of the healthy body (Carel, 2016, p. 55). Rather, the ill person’s body can no longer escape their attention as it gains a certain opaqueness. This perpetual focus on the body is often further exacerbated by the clinical treatment of somatic illness. Under the medical gaze, the patient is objectified as their body is tested and measured in comparison to the “healthy” body. These bodily measurements often become incorporated into the patient’s daily lives, as they are required to monitor their heart rate, blood sugar levels, peak expiratory flow and so on. This puts a greater emphasis on *having* a body rather than *being* a body for the ill subject.

Carel distinguishes between the somatic and mental forms of infringement upon one’s motility as follows: “illness can destroy creativity in one of two ways: either by removing the capacity to fantasize or by removing the capacity to execute” (Carel, 2016, p. 73). Carel understands “removing the capacity to execute” as a physical inability to perform certain bodily actions. In contrast, she recognizes the breakdown in the ability to “fantasize” bodily motility to be prominent in psychiatric illness. Although this infringement is not rooted in the body in the same way, people with psychiatric conditions may experience the environment transform from familiar to foreign, as the world no longer offers possibilities for engagement in the way it once did.

People diagnosed with depression, for example, report difficulties in performing the most everyday habitual actions, such as making a cup of tea or crossing the room: “it takes an enormous amount of effort to engage with the world and your own life” (cited by Ratcliffe, 2015, p. 33).^2^ In cases of agoraphobia, the illness imposes upon the person an inability to leave the realm of “home” or the familiar: “the centrality of the physical home, with its borders and boundaries, marks a threshold from agoraphobic embodiment to non-agoraphobic embodiment” (Trigg, 2013, p. 418). As one patient with schizophrenia describes: objects in the world “[seem] so far away as if there is an invisible wall I cannot penetrate” (Krueger et al., 2016, p. 260). Here the ill subject is unable to grasp the meaning of their environment and thus struggles to “imagine” the body’s engagement with the world.

The shift in the patterns of embodiment produced by illness influences the subject’s sense of belonging to the world. On this basis, Carel claims that meaning and intelligibility depend on consistent patterns of embodiment. When these patterns are disrupted, meaning is affected’ (Carel, 2016, p. 15). This disruption is particularly dramatic in psychiatric illness (ibid). Merleau-Ponty demonstrates this as he places the dynamic, meaningful and cohesive scene of Paris in stark contrast with the ambiguous landscape experienced by someone with schizophrenia:

Suddenly the landscape is snatched away from him by some alien force. It is as if a second limitless sky were penetrating the blue sky of the evening. This new sky is empty, “subtle, invisible, and terrifying.” Sometimes it moves into the autumn landscape, and sometimes the landscape itself moves … The schizophrenic patient no longer lives in the common world but in a private world; he does not go all the way to geographical space, he remains within “the space of the landscape,” and this landscape itself, once cut off from the common world, is considerably impoverished.

(Merleau-Ponty, 2012, p.300).

Merleau-Ponty demonstrates that psychiatric illness constitutes a breakdown in the meaning structures of a person’s world. What once appeared as part of a meaningful whole, say a clock, no longer speaks to the embodied subject in the same way. In the words of Merleau-Ponty, he can no longer “understand” the clock: “first the passing of the hands from one position to another and above all the connection of this movement with the thrust of the mechanism or the ‘workings’ of the clock” (Merleau-Ponty, 2012, p. 295). There is no longer a cohesive whole of “clock” or “world” for the schizophrenic person.

This lack of cohesive whole, in turn, impacts how objects in the world invite interaction. For instance, Krueger explains that commonly in schizophrenia:

People and things are no longer encountered as “ready-to-hand”^3^—as affording a range of immediately perceived interactive possibilities (the way a friendly smile affords conversation or a chair sitting) specified by the norms and conventions tacitly governing the context in which they’re encountered. Instead, everyday encounters and projects are experienced as puzzling or devoid of meaning

(Krueger, 2020, p. 602).

So too, in depression, the subject commonly encounters what Ratcliffe terms a “severed reality”: “the depressed person finds herself in a different ‘world’, in an isolated, alien realm that is cut off from the consensus reality” (Ratcliffe, 2015, p.15). Aho says of anxiety that “nothing stands out as significant anymore; my job, my relationships, my commitments, the very things I rely on to construct a coherent and unified life-story, are stripped of their import. And this undercuts my own ability ‘to be’” (Aho, 2020, p. 8). Through this “unworlding”, a distance emerges between the subject and the world as the patterns of embodiment that serve as a backdrop to the person’s very existence collapse.

## Unworlding & language

Elaine Scarry says of pain: “for the person whose pain it is, it is ‘effortlessly’ grasped (that is even with the most heroic effort it cannot not be grasped); while for the person outside the sufferer’s body, what is effortless is not grasping it” (Scarry, 1985, p. 4).^4^ The suffering’s “resistance to language” is apparent in numerous pathographies, including ones written by people with psychiatric illness (ibid). In *Darkness Visible*, William Styron describes depression as “so mysteriously painful and elusive in the way it becomes known to the self … as to verge close to being beyond description. It thus remains nearly incomprehensible to those who have not experienced it in its extreme mode” (Styron, 2010, p. 5). So too, in describing her experience of bipolar disorder, Nancy Tracey claims emotional pain is even harder to express than physical pain:

Language is insufficient to express emotional pain and turmoil. We have good words for describing physical pain: radiating, hot, throbbing, sharp, achy and so on. But when it comes to emotional pain we’re “sad.” … It’s not surprising that people don’t get what we’re talking about

(Tracy, 2016).

Scholars in the phenomenology of illness have attributed this communication breakdown to the fundamental taken-for-granted elements of the world being drastically altered for the person with a psychiatric illness. Due to a monumental shift in the ill person’s embodied experience, she is thrust into an unfamiliar life-world with new, confusing, and inexpressible meaning-structures. As such, people with psychiatric illness become “experientially unmoored from the lived spaces of their everyday environments” (Krueger, 2020, p. 602). It appears that the inexpressibility of illness is driven by an “unworlding”, whereby the ill person experiences a disruption between self and world due to an inability to orient themselves in the now alien environment.

After observing the extreme difficulty, or even inability, people with depression experience when trying to put their experience into words, Ratcliffe argues:

Sometimes, this difficulty is no doubt partly attributable to effects that depression has on one’s cognitive abilities. But people still struggle to convey the experience after recovering, and their accounts often suggest that the problem stems from its very nature. Depression involves a disturbance of something that is fundamental to our lives, something that goes unnoticed when intact. What is eroded or lost is a “sense” or “feeling” of being comfortably immersed in the world.

(Ratcliffe, 2015, p.16).

Thus, the difficulty in expressing psychiatric illness can be understood as a product of one’s profoundly altered structure of experience, also known as an epistemically “transformative experience” (Paul, 2014). Illness “gives us experiences that we would not otherwise have had and that we cannot know what it is like to have until we undergo them – knowledge that cannot otherwise be acquired” (Carel et al., 2016, p.1152). In other words, certain experiences, such as childbirth or an ecstatic religious experience with a God, can only truly be understood by those who have had both “the requisite bodily experience” (as in the case of childbirth) and, or, the requisite interpretation of the world (for instance, an ecstatic religious experience requires an understanding of the world as one with a God) (Kidd & Carel, 2017, p.185). Take depression; according to Styron, the incomprehension of the illness by others is driven not by a lack of sympathy, “but the basic inability of healthy people to imagine a form of torment so alien to everyday experience” (Styron, 2010, p.14–15). Interchanging pain for illness more broadly, it would seem that illness “does not simply resist language but actively destroys it, bringing about an immediate reversion to a state anterior to language” (Scarry, 1985, p. 4).

Thus, much of the current literature in phenomenological psychopathology concludes that an inability to find oneself at home in the world leads to a breakdown in language. However, an examination of the work of Sass reveals that the relationship between language and “unworlding” may be more complicated than previously assumed. In “Madness and Modernism” (2017), Sass explores this language breakdown as it relates to people with Schizophrenia (Sass, 2017, p. 141–169). People with schizophrenia are met with “acute experiences of the inadequacy of language” whereby words no longer seem to possess the hermeneutical power to signify objects and concepts in the world. Sass associates this ineffability with a tendency toward “a poverty of speech” (Sass, 2017, p.153). Due to the unusual perceptions common in schizophrenia, many patients turn away from “social imperatives and realistic concerns” that our language is equipped to articulate and become preoccupied instead with a reflection on inner phenomena (Sass, 2017, p. 169). As it stands, at least in Western cultures, our vocabulary for the experience of “inner phenomena” is inadequate. As such, a central factor of communication breakdown in schizophrenia is due to a newfound focus on some of the most ineffable aspects of our experience. Briefly, Sass even suggests that this increased attention on inner phenomena may, in fact, “create” the “detached scrutiny, introversion and disengagement” common in schizophrenia (Sass, 2017, p. 152).^5^ Sass implies that the inability to articulate inner phenomena may cause the experience of unworlding.

This suggests that the relationship between the experience of “unworlding” and ineffability may not be unidirectional. While the experience of “unworlding” may drive a “poverty in speech” in psychiatric illness, the “poverty in speech” may, in turn, drive the experience of “unworlding”. Through this paper, I bring Sass’s observation to the fore and take it beyond schizophrenia to examine ineffability in psychiatric illness more broadly. I challenge the unidirectional causal relationship that depicts the experience of “unworlding” as driving the loss of speech expression in psychiatric illness. I identify a two-way causal relationship between the collapse of the ill person’s being-in-the-world and the breakdown in speech expression.

Moreover, while Sass suggests that a “poverty of speech” is a likely outcome of attempting to articulate the most complex and inaccessible of experiences, experiences that our vocabulary is ill-equipped to express, I argue that “poverty of speech” in psychiatric illness may also derive from epistemic injustice. More precisely, it is a hermeneutical injustice. Although “unworlding” can obstruct the voice of the ill person, external strategies of hermeneutical injustice can sustain and even further perpetuate the experience of unworlding. As we shall see, the voice of those with psychiatric illness can be obstructed not only by an altered being-in-the-world but also by socially embedded epistemic practices. Hermeneutical injustice is an external force that has the power to drastically curtail the speech expressions of those with psychiatric illnesses. To fully understand the role “speech poverty” plays in the experience of psychiatric illness, we need a phenomenological account of speech expression. For that, I turn to Merleau-Ponty.

## Merleau-Ponty on speech expression

### Speech & the body

Imagine immigrating to a foreign country without knowing the language. You arrive to find the environment around you appear strange and unfamiliar. You are struck by uncanny gestures, features of the landscape and objects in your vicinity that those at home in this country pass by unnoticed. While you are accustomed to confidently moving through your environment with ease, you move with caution and heightened attention in this foreign country as you navigate this unfamiliar terrain. What’s more, you are painfully aware that this environment is meaningful to others around you, those who reside in the area. Thus, you have an acute sense of not belonging to this world. Initially, this observation may appear philosophically uninteresting: of course, you would be unable to identify certain objects due to their cultural signification. Nor would you be able to communicate with others, thus restricting your ability to form interpersonal relationships. However, say these particular hurdles are overcome by acquiring a comprehensive guide to the culture and acquiring a book of translation; I suggest you would still be unable to fully belong to this world because “*being-in-the-world”* requires embodying the language of that world.

So, what makes language meaningful? I believe we can find the answer in the following observation by Merleau-Ponty:

The full sense of a language is never translatable into another. We can speak several languages, but one of them always remains the one in which we live. In order to wholly assimilate a language, it would be necessary to take up the world it expresses, and we never belong to two worlds at *the same time*

(Merleau-Ponty, 2012, p. 193, my italics).

In line with Merleau-Ponty, I propose that to “wholly assimilate” (to truly live) a language requires a “taking up of the world”, a dwelling in the environment. Language is the core structure of our being-in-the-world. It is a form of bodily comportment that discloses the meaning of the world and is, therefore, central to our ability to move through our environment with a pre-reflective openness. For this reason, the meaning of one language can never be fully translatable to the meaning of another as this would require existing in two different worlds *simultaneously*.

To argue that a person could be bilingual or multilingual and therefore belong to more than one world due to their immersion in multiple languages would be to misunderstand Merleau-Ponty. Rather, Merleau-Ponty argues that a person cannot *simultaneously* dwell in more than one world. Like the duck-rabbit illusion made famous by Wittgenstein (where one can never see the duck and rabbit simultaneously), a person can only be immersed in one linguistic world at a time (Wittgenstein, 2009, p. 400). Drawing on the work of Heidegger, Merleau-Ponty has introduced a theme which will play a central role in his phenomenology: our “being-in-the-world” is essentially linguistic.

Merleau-Ponty confronts the intellectualist account of speech expression, which rests on a misunderstanding of the relationship between thought and speech. In line with common assumption, the intellectualist adopts the mistaken idea that speech is internally pre-expressed through thought. As Merleau-Ponty portrays it, intellectualism assumes that within this inner realm meaning is made and then translated into speech. In Merleau-Ponty’s rejection of this account, he considers the experience of inner speech to be a possible culprit for this common assumption as it prompts the “illusion of an inner life” (Merleau-Ponty, 2012, p.189). Instead, Merleau-Ponty claims that there is no thought prior to speech expression other than “the muted language in which being murmurs to us” (Merleau-Ponty, 1973, p.6). The formless murmur, “buzzing with words” lingers prior to speech as a pregnant source of potentiality (Merleau-Ponty, 2012, p.189). These “murmurs” are made meaningful only when accomplished through speech expression. There is a certain urgency in expressing this ambiguous “buzz of words” public. It is effectively the elusive traces of a forthcoming speech expression. Thus, the word is no mere vessel for thought but the external accomplishment of thought.

Thought is an act accomplished not only through speech expression but through all forms of bodily expression. For Merleau-Ponty, a gesture is not superfluous to thought, or a mere additional flourish. Rather, gesture *is* thought: “the gesture does not make me think of anger, it is anger itself” (Merleau-Ponty, 2012, p. 190). No inference is necessary. This is not to be mistaken for the claim that to observe an instance of anger, through the shaking of a fist, for example, is equivalent to having a first-person experience of another person’s anger. That would result in an inability to distinguish between the subjective “I” and the “Other”. In the words of Gallagher and Zahavi, the gesture is “saturated with the meaning of the mind; it reveals the mind to us”, and in this sense, we experience the emotion as directly as we can without first-person access (Gallagher & Zahavi, 2012, p. 207). Therefore, gesture does not merely signify meaning *but is meaning itself*. Before gestural expression, the gesture lingered in the subject’s mind as a vague fever and only through expression can it achieve reality.

With a Merleau-Pontian account of gesture established, we can make our way toward an understanding of speech expression as a form of bodily gesture. Initially, it may appear trivial to identify speech as bodily. Speaking is, of course, a corporal act as it requires vocal cords, amongst other bodily functions to be carried out. More significantly, however, speech expression is a way I can employ my body to engage with the world. Speech expression is part of the “body schema”: the possibilities of my body, through which I can interact with the complex tapestry of meaning in the world. By uttering its name, we reach toward objects in the world, bring them to life and make them tangible. Thus, as a form of bodily expression, speech holds gestural meaning: “speech is a gesture, and its signification is a world” (Merleau-Ponty, 2012, p. 190).

### Speech & others

Merleau-Ponty urges that we operate within a world that we understand to be shared with Others. We do not experience Others in the world in the same way as we do other objects. We witness that Others in the world must possess a subjectivity like my own, because they engage with the world in much the same way as myself.^6^

Merleau-Ponty exemplifies intersubjectivity by observing a man lying in the sun (Merleau-Ponty, 2012, p. 184). Merleau-Ponty recognizes that he and the other man share a common world as they are simultaneously impacted by a significant aspect of the world: the sun burns them, makes their eyes squint, makes them sweat and makes them raise their hand over their forehead in a protective gesture or reach for a hat. Merleau-Ponty identifies in the Other’s gestures that they experience the same “bite of the world” (Merleau-Ponty, 1973, p. 137). This symmetry convinces him that the Other is moved and touched by the same world, and thus, is an embodied subject positioned in the world much like himself. Moreover, he is aware of himself as a subject in the eyes of the Other, as they too witness Merleau-Ponty as a being impacted by the sun: “I feel that someone feels me, that he feels both my feeling and the very fact that he feels me” (Merleau-Ponty, 1973, p. 135).

Merleau-Ponty does not merely suggest that we have an awareness of the Other. The Other constitutes our own subjectivity to some extent, and to some extent we constitute theirs. Merleau-Ponty does not suggest that it is possible to genuinely constitute Others in the manner they constitute themselves. This would lead us to the dilemma of being unable to distinguish between an “I” experience and an “Other” experience. Rather, we embrace the Other into our body schema in the same way we embrace other aspects of the world. For Merleau-Ponty, the body schema is, in part, constituted by objects in the world. The pen is part of my bodily possibilities to write; the tea is part of my bodily possibility to drink, and so on. Similarly, the body schema embraces the Other as a means for interaction. When we see the gesture of the Other frowning in anger, we do not infer anger from their gesture but witness it directly: “It is the simple fact that I live in the facial expressions of the other, as I feel him living in mine. It is a manifestation of what we have called, in other terms, the system ‘me-and-other’” (Merleau-Ponty, 1964, p.154). Here, Merleau-Ponty refers to the bodily aspect of intersubjectivity, which is known as *intercorporeality*.

Thus, we can see how the intercorporeality of “I” and “Other” is a fundamental aspect of successful dialogue. When we encounter the speech gesture of the Other, we “take up” their meaning “in so far that this is possible given the differences between our bodies, our histories, and the modes of expression” (D. Landes, 2013, p. 92). By “taking up”, Merleau-Ponty refers to the body adjusting to the speech gesture of the Other and encompassing it into its infrastructure, adding to its “evolving weight” and gearing it toward the world (ibid). This is what makes for a successful dialogue. Rather than undergoing a process of translating a word to an idea, this “taking up” occurs instantaneously: “there is a taking up of the other person’s thought, a reflection in others, a power of thinking according to others, which enriches our own thoughts” (Merleau-Ponty, 2012, p.184).

### Fanon

Recall Merleau-Ponty’s bold claim that we can never truly *speak* the language of a foreign world because we can only “live” one linguistic institution at a time. As Landes puts it: “To understand English is not to ‘possess’ it in my mind in some mental lexicon. Rather, speaking English involves having English gestures ready-to-hand” (D. Landes, 2013, p. 134). Drawing on the work of Frantz Fanon, I suggest that Merleau-Ponty’s analogy of the foreigner betrays more than he intends. The philosopher Fanon shares several similarities to Merleau-Ponty. He was a French-speaking existentialist, heavily influenced by psychoanalysis, a veteran of the second world war, and published his work during a similar time frame to Merleau-Ponty (1952–1961). In moving from the French colony of Martinique to France around 1945, Fanon became the “foreigner” in Merleau-Ponty’s analogy. *Black Skin, White Masks* (1952) begins with an exploration of the phenomenology of language. Like Merleau-Ponty, Fanon recognized that “To speak means being able to use a certain syntax and possessing the morphology of such and such a language, but it means above all assuming a culture and bearing the weight of a civilization” (Fanon, 2008, p. 1). Indeed, he identifies that to possess a language is to possess the world of this language. Yet, for Fanon, to be a speaking subject is not the universal gift that Merleau-Ponty presents it to be.

Fanon describes the experience of the black “creole” (pidgin French) speaking Antillean who moves to France and attempts to assimilate the “proper” French language. He does so, according to Fanon, because “proper” French is (mistakenly) regarded as the golden ticket that grants permission to a white world: “the more the black Antillean assimilates the French language, the whiter he gets- i.e., the closer he comes to becoming a true human being” (Fanon, 2008, p. 2). Creole is given so little credibility that it is barely considered a language at all, banned from some households for being “vulgar”. Fanon places creole in stark contrast to what he calls the refined “white” version of French: “The French from France, The Frenchman’s French, French French” (Fanon, 2008, p. 4). The Antillean consequently rejects creole, moves to Paris and becomes fluent in “proper” French with the intent of *belonging* to France, “i.e. the real world” (Fanon, 2008, p. 20). But, Fanon observes, the Antillean man has been duped. The golden ticket can never be attained. Even if he becomes fluent in “proper” French, he is structurally barred from belonging to their world. He is not assimilated as another “speaking subject” in the way Merleau-Ponty depicts because, in virtue of his race, he is prohibited from the status of “speaking subject”. His speech expression does not receive the same uptake due to his race. As such, in France, he is inhibited from living the language that allows him being-in-the-world.

Fanon helps us expose a gap in Merleau-Ponty’s phenomenology of speech expression as this phenomenological account is limited to those granted a hermeneutical privilege. Only those in a dominant social position have the status as “speaking subject”, and the subject-world synthesis that accompanies it. Fanon limits his analogy to all persons who are colonized. Still, it would not be a stretch to argue that other features of one’s embodiment (one’s race, sexuality, age, gender, ability, and so on.) has the power to inhibit one’s membership to the community of speaking subjects.

What Fanon identifies can be termed “Epistemic injustice”. Epistemic injustice was first theorized by Fricker to “delineate a distinctive class of wrongs, namely those in which someone is disingenuously downgraded and/or disadvantaged in respect of their status as an epistemic subject” (Fricker, 2017, p. 53). The epistemic nature of the injustice derives from a person being wronged in their capacity as a knower. In the example presented by Fanon, the Antillean does not register as a “knower” to the French, as someone worth listening to or taking seriously. While there are many harms at play here, in relation to language, this can be identified as a specifically epistemic harm. Fricker distinguishes two forms of epistemic injustice: hermeneutical injustice and testimonial injustice. This paper will focus on the former. In what follows, we will focus on the precarious hermeneutical position of the psychiatric patient.

## The breakdown of speech expression

### Hermeneutical injustice

To exemplify hermeneutical injustice, Fricker presents the case of victims of sexual harassment prior to the 1960s. Because certain groups most likely to be targeted by sexual harassers were excluded from the construction of interpretive frameworks in the workplace, experiences of sexual harassment were not part of the collective understanding. Instead, “repeated sexual propositions in the workplace [were] never anything more than a form of “flirting”, and their uneasy rejection by the recipient only ever a matter of her lacking a ‘sense of humor” (Fricker, 2007, p.152–153). Given this “hermeneutical lacuna”, to use Fricker’s terminology, victims of sexual harassment were hindered from articulating the harm inflicted upon them. Consequently, victims were not only unable to report or discuss sexual harassment effectively, but they also lacked the hermeneutical resources required to fully grasp the experience themselves.

It is worth adding that a total gap in the hermeneutical resources like that described above is a rare occurrence. Although Fricker uses terms such as “lacuna” or “gap”, I suggest that it is better to consider the “pool of shared ideas” (to borrow Fricker’s metaphor) as being more or less depleted (and in rare cases, there may even be a complete drought). Only the hermeneutically privileged can contribute toward, alter, and remove resources from the pool of shared ideas. The hermeneutically marginalized do not have this power and are forced to contend with ill-fitting concepts.

Two harms emerge from hermeneutical injustice. The first is an epistemic harm, whereby the marginalized subject is undermined as a knower. The secondary harm Fricker identifies is that of “cognitive disablement”:

The cognitive disablement prevents her from understanding a significant patch of her own experience: that is, a patch of experience which it is strongly in her interests to understand, for without that understanding she is left deeply troubled, confused, and isolated, not to mention vulnerable to continued harassment

(Fricker, 2007, p. 151).

Fricker understands hermeneutical resources as essential for meaning-making; where hermeneutical resources are missing from the interpretive framework, the subject’s grasp of their experience is distorted, limited, or otherwise confined.

### Hermeneutical injustice in psychiatry

In the latest “Big Mental Health Survey” conducted by Mind, 86% of participants reported experiencing discrimination in at least one life area.^7^ The survey showed high levels of discrimination reported in the participant’s social life, employment, education and online. Such stigma and prejudice directed at those with psychiatric illness have been dubbed “sanism”, through which people are discriminated against and oppressed in virtue of their psychiatric illness. Like racism or sexism, Michael L. Perlin popularized the term “sanism” to draw attention to the discriminatory distinction between the “mad” and the “sane” (Perlin, 1992).^8^ This distinction has far-reaching philosophical roots in the work of Michel Foucault, who formulated a genealogy of “madness” to trace back the schism that separated the so-called “man of madness” from the “man of reason” (Foucault, 2001). In the preface to *Madness and Civilization*, Foucault defends the urgent need for such a genealogy due to the breakdown in communication between these two groups:

The constitution of madness as a mental illness … affords the evidence of a broken dialogue, posits the separation as already affected, and thrusts into oblivion all those stammered, imperfect words without fixed syntax in which the exchange between madness and reason was made. The language of psychiatry, which is a monologue of reason about madness, has been established only on the basis of such a silence.

(Foucault, 2001, p.xii).

For Foucault, the entrenched sanism in our society is driven by a disparity between the voice of the “mad” and the voice of the “sane”. Specifically, the voice of the “mad” and the voice of the psychiatrist.^9^

Healthcare professionals attempted to overcome the inevitable communication barriers posed by psychiatry through the development of a universal psychiatric vocabulary. This universal vocabulary took the form of diagnostic manuals, the most popular today being the *Diagnostic and Statistical Manual of Mental Disorders* (DSM). According to the APA, the aim of the latest edition of the DSM is as follows:

[to create] a common language for clinicians to communicate about their patients and [establish] consistent and reliable diagnoses that can be used in the research of mental disorders. It also provides a common language for researchers to study the criteria for potential future revisions and to aid in the development of medications and other interventions

(DSM-5).

Whilst the DSM may go some way toward creating a universal framework for understanding psychiatric illness amongst healthcare professionals and researchers, what is missing from this mission statement is the pursuit of a common language between clinician and *patient*. By emphasizing communication *about* patients rather than *to* patients, the APA suggests that the patient’s understanding of their psychiatric illness is secondary to that of the clinician and researcher.

Thus, despite the central role the doctor-patient dialogue plays in psychiatry, the voice of the patient is notably omitted from diagnostic manuals. Instead, an epistemic privilege is afforded to the third-person perspective of the DSM and the psychiatrist who mediates it: “the medical perspective is regarded not only as authoritative but often even exclusive of other perspectives, such that medical diagnosis effectively constitutes a monopoly on the way the experience is interpreted” (Scrutton, 2017, p.349). As a result, the sense-making of psychiatric illness has been limited to that which can be conveyed through the language of the DSM.

The purpose of the DSM is to provide an accurate description of psychiatric illnesses that mirrors the experience of those with them. Therefore, it is reasonable to suggest that third-person insight and empirical data alone cannot create the robust diagnostic criteria needed for an accurate diagnosis. Consider the case-study put forward by Ratcliffe et al., comparing the symptoms of a bad case of the flu to the diagnostic criteria for major depressive disorder (Ratcliffe et al., 2013). According to the DSM-5, to be diagnosed with major depressive disorder, the person must display at least five out of nine symptoms, such as “significant weight loss”, “decrease … in appetite”, “fatigue or loss of energy”, “diminished ability to think or concentrate”, “markedly diminished interest or pleasure in all, or almost all, activities most of the day” and “psychomotor agitation or retardation” (DSM, 2013, p.160–161). Ratcliffe et al. conclude:

Given the phenomenologically permissive way in which depression is described by diagnostic systems … the general feeling of being unwell, associated with illnesses such as influenza, does indeed meet the criteria for a major depressive episode, at least in those cases where another illness has not been diagnosed.

(Ratcliffe et al., 2013, p. 206).

Ratcliffe et al.’s study demonstrates a deficiency in the hermeneutical resources where the patient’s first-person experience is obscured. By excluding the first-person account of psychiatric illness from diagnostic criteria, we are left with a picture of major depressive disorder that is experientially the same as the flu. To better distinguish between these two symptomologies and to create a more accurate account of illnesses like depression, the patient’s first-person insight ought to be encompassed within the diagnostic criteria. After all, “the people who might best know the various subtleties of a disorder and the criteria that could best be used to describe them are those who have first-hand experience with that disorder on a daily basis” (Flanagan et al., 2010, p. 303).

In response to Sadler et al.’s question, “Should Patients and Their Families Contribute to the DSM-5 Process?” (Sadler & Fulford, 2004), Spitzer (chair of the task force behind the DSM-3) reacts as if even raising this question is to attack the moral standing of the psychiatrist:

It is insulting to the mental health professionals involved in the DSM revision process, many of whom have family members with psychiatric illness or have experienced illness themselves, to suggest that they are insensitive to such issues and that they need to be educated by patients and families

(Spitzer, 2004, p. 113).

Spitzer goes on to argue that it is “politically correct nonsense” to suggest that psychiatric patients and their family members could provide a unique insight into diagnostic criteria that “committees of mental health professionals who are chosen because of their expertise in some aspect of psychiatric diagnosis” could not possess (ibid).

The upshot of such hermeneutical injustice can be found in Stanghellini and Mancini’s analysis of the “structured clinical interview” or “technical interview”, the interviewing method developed by Spitzer for assessing psychiatric symptoms. The structural clinical interview, combined with the diagnostic criteria of the DSM-5, make up the diagnostic process Stanghellini and Mancini refer to as “the technical approach” (Stanghellini & Mancini, 2017). Spitzer set the structured interview apart from the previous diagnostic models by limiting the variance of patient responses collated through the interview process: “Information variance was minimized by the use of a structured interview that ensured that the clinician systematically covered all the relevant areas of psychopathology” (Spitzer, 1983, p. 401). In reducing the variance of patient responses, Spitzer aimed to improve the accuracy and reliability of the diagnosis.

Consequently, in “reducing the variance of information”, Spitzer’s interview process was designed to omit any questions that may produce answers that were seemingly “irrelevant” to the diagnosis: “Obviously, the relevance of some phenomena (and the irrelevance of all the others) is decided *a priori* – i.e., before the interview with that singular person takes place. The consequence is that a great deal of abnormal phenomena may pass unobserved” (Stanghellini & Mancini, 2017, p. 9). The structural interview avoids asking questions concerning areas of the patient’s first-person experience that may be deemed irrelevant, e.g., “manifold disturbances of embodiment, lived space, and time” (Stanghellini & Mancini, 2017, p. 8). This leads to a “performatively produced” hermeneutical injustice, whereby an expressive style is excluded from the interpretive framework (Medina, 2017, p. 45–46).^10^

Following Fricker’s account, the unequal hermeneutical participation of a marginalized group from “some practice that would have value for the participant” (in this instance, the development of the language of psychiatry) leaves a depleted pool of hermeneutical resources (Fricker, 2007, p. 153). Eliminating the voice of those with psychiatric illness from diagnostic manuals not only restricts the content and accuracy of diagnostic categories but also constitutes an injustice against the marginalized subject, as it prevents them from contributing toward the creation of hermeneutical resources vital for expressing their experiences. In line with Fricker, the subject is met with a cognitive dissonance, whereby their personal experience of psychiatric illness is at odds with the language proposed by the DSM. The following section will explore the impact of hermeneutical injustice on one’s capacity for meaning-making in psychiatric illness.^11^

## The phenomenological impact of hermeneutical injustice

### Hermeneutical injustice & the body

To understand the phenomenological impact of hermeneutical injustice in psychiatric healthcare, let us turn to Merleau-Ponty’s concept of “movement toward the possible” (Merleau-Ponty, 2012, p.109). For Merleau-Ponty, when a subject successfully performs an action, there is no gap between the intention to act and the action itself. For example, when a footballer throws her body into the action of kicking a ball, there is a harmonious and invisible bond between the “intentional threads” that pull her toward kicking the ball and the actual action of kicking (Merleau-Ponty, 2012, p.108). Now imagine the footballer with phantom limb syndrome. Upon seeing the ball, it still offers the footballer the same intentional threads as before, suggesting to her possibilities for action. Although the subject feels the pull of intention toward action, the action is stunted as she is missing an essential feature of her body schema to perform this action.

In virtue of being a speaking subject, the hermeneutically marginalized feel a habitual pull toward speech expression as “the intention to speak can only be found in open experience: it appears, as boiling appears in liquid, when in the thickness of being, empty zones are constituted and move outwards” (Merleau-Ponty, 2012, p. 202). Although the hermeneutically marginalized is pulled toward an act of speech expression, they are confronted by an absence in their body schema where the hermeneutical resource ought to be. With scarce resources to describe their experience, the person with psychiatric illness is thrown into a paradoxical state, whereby the habitual body anticipates the capacity for speech expression yet is met with a negation in the phenomenal field. Thus, the hermeneutical injustice elicits an experience of *embodied* dissonance, whereby the hermeneutically marginalized experiences a divide between body and world, eliciting an “unworlding”.

As previously discussed, this experience of unworlding is characteristic of psychiatric illness as those thrust into a state of psychiatric illness are forced to reexamine the way they encounter the world. Actions pre-reflectively performed by the habitual body, such as getting out of bed or making a cup of tea, cannot be accomplished without explicit attention (if they can be accomplished at all). In the words of Styron, “I began to experience a vaguely troubling malaise, a sense of something having gone cockeyed in the domestic universe I’d done so long, so comfortably” (Styron, 2010, p.41). The literature on the phenomenology of psychiatric illness broadly focuses on the profoundly altered structure of experience, “where the absence of hope, practical significance, and interpersonal connection is painfully felt” (Ratcliffe, 2015, p. 55).

Without speech expression, the person with psychiatric illness cannot throw their body into an act of free and open expression in the same way as their hermeneutically privileged counterparts. For example, in recounting his vain attempts to communicate with his psychiatrist, Styron describes such phenomenological deterioration in his speech expression: “my speech, emulating my way of walking, had slowed to the vocal equivalent of a shuffle” (Styron, 2010, p. 55). When psychiatric patients like Styron attempt to put into words their experience of psychiatric illness, they are often stunted. Speech expression is no longer an invisible act but one at the forefront of the person’s attention as they fumble over ill-fitting hermeneutical resources.

Without the capacity to express one’s illness experience, the hermeneutically marginalized lose an essential way they were tied to the world. Unable to exploit the hermeneutical resources that once rolled off the tongue, engagement with their environment is strained. The environment no longer invites interaction in the way it once did. In this sense, the hermeneutically marginalized subject suffers an “unworlding” as the look and feel of the world is altered. Through a phenomenological perspective, it becomes clear that the unequal hermeneutical climate of psychiatric healthcare perpetuates, and even exacerbates, this experience of “unworlding” for the person with psychiatric illness.

The world-altering nature of hermeneutical injustice can be found in Steslow’s essay “Metaphors in Our Mouths: The Silencing of the Psychiatric Patient”, where Steslow describes her stint of involuntary incarceration in two psychiatric institutions. In reflecting upon her experience in psychiatric care, Steslow reports: “what I found most distressing – what threatened to erode any composure I could manage in hospital – was not the involuntary commitment, *but rather the distinct feeling of being unheard”* (Steslow, 2010, p. 30, my italics). Once within the confines of a psychiatric institution, she “was cut off from all meaningful conversation by the veil of [her] diagnosis”; her speech expressions no longer carried the same weight, as everything she said was perceived to be a product of her illness (ibid). In her fight to be heard, Steslow was forced to adopt the medical terminology of the psychiatric experts for her speech to be considered meaningful. As such, she molded her speech expression to fit within the confines of the restrictive medical framework:

There was a clear and distinct vocabulary being used to talk about my experience, and that vocabulary was not mine. But by adopting it, I began to regain some standing as a speaker worth listening to; I was then judged to exhibit that peculiarly esteemed quality psychiatrists call insight

(Steslow, 2010, p. 30).

To gain credibility, Steslow was forced to adopt ill-fitting hermeneutical resources, “forsaking the uniqueness of [her] own perspective, understanding, and expression”, in the hope that she would be heard in some capacity (ibid).

Thus there is a distinct hermeneutical injustice that prevents her from being able to talk about her illness in her own terms. While the purpose of the psychiatric framework is to render the patient’s narratives intelligible in a medical context, “much of its healing power is lost in the wake of alienation, dis-empowerment, and silencing” (Steslow, 2010, p. 30). Through the language of psychiatry, Steslow could only understand her experiences (to use Fricker’s phrase) “through a glass darkly”, as the interpretative framework did not correlate with her own experience (Fricker, 2017, p.148):

A gulf widened between the self I was able to be outside the hospital and the self I had to present inside. I spoke as I knew I had to in order to be heard, aware of the dishonesty that saturated every obeisance and *distressed that I was losing a sense of wholeness*, splitting apart the young woman whose religious and existential crises had precipitated a desperate self-assault and the young woman who pretended that group therapy was interesting and helpful in order to move a notch further toward her discharge

(Steslow, 2010, p. 30, my italics).

Unable to authentically express herself, Steslow describes a loss of “a sense of wholeness” (ibid). As Steslow observes, oppressive hermeneutical practices that force psychiatric patients to mimic a remote medical voice “may end by creating minds more fragmented in *perceiving* and speaking than those that first turned up for help” (Steslow, 2010, p. 30). Yet, rather than “minds” what Steslow truly identifies here is a “splitting apart” of an embodied being, as the cohesiveness of her being-in-the-world is interrupted by hermeneutical injustice.

### Hermeneutical injustice & the other

A further consequence of hermeneutical injustice is a loss of not only one’s capacity for an essential form of embodied expression, but also a loss in one’s intersubjectivity. A breakdown in intersubjectivity is a common feature in psychiatric illness. For example, in depression “an overarching theme is that a type of interpersonal connection, which most people take for granted in the course of everyday life, no longer seems possible” (Ratcliffe, 2018, p. 1). In agoraphobia “what is peculiar to the agoraphobic experience of others is a conflict between the personal experience of the body and the impersonal relationship the body has to others” (Trigg, 2013, p. 414). In schizophrenia, a necessary feature is “disturbances of the intercorporeality with others, with subsequent disconnection from the social environment” (Fuchs & Röhricht, 2017, p. 128). While this breakdown in intersubjectivity may result from the illness itself (Pienkos, 2015; Sass, 2017), it is exacerbated by hermeneutical injustice.

Recall Merleau-Ponty’s example of the man in the sun, whose gestures demonstrate that he engages and is affected by the world in the same way as the observer. From this, Merleau-Ponty recognizes that the man is an embodied agent in the world, much like himself. So too, when the hermeneutically privileged witness the Other perform speech gestures like their own and reference the world in the same way they do, they understand that they belong to a shared world with this speaking subject. The hermeneutically privileged make their speech expression against the background of this shared world: “the verbal gesture must be performed in a certain panorama that the interlocutors share, just as the comprehension of other gestures presupposes a shared world shared by everyone in which the sense of gesture unfolds and is displayed” (Merleau-Ponty, 2012, p.200).

Drawing on Merleau-Ponty, we can concede that forming a “social whole” with the Other through dialogue is an essential aspect of one’s being-in-the-world. Those subjected to hermeneutical injustice are robbed of this intersubjectivity.^12^ Speech expression is set against a background of shared collective understanding, cemented by those in a dominant social position into “the alphabet of acquired significations”; a shared world in which the interests of the powerful, rather than the powerless, are perpetuated. This sedimented collective understanding allows one to take up the speech expression of the Other. If one’s required hermeneutical resources are absent from the collective understanding, there is a breakdown in the I-Other dialogue whereby “the other who listens and understands joins with me in what is most singular in me” (Merleau-Ponty, 1973, p. 141). As their speech expressions are born from the same linguistic institution, there is an overlap in the distinct body schemas of each hermeneutically privileged subject. This is known as intercorporeal. Those inflicted with hermeneutical injustice cannot participate in this overlapping of body schemas of the Other.

### Hermeneutical justice and talking therapies

The phenomenological impact of hermeneutical injustice in psychiatric healthcare can be further exemplified through the success of talking therapies. A Gadamerian approach to psychiatry promotes “the art of healing”: using doctor-patient dialogue to grasp the disturbance in the patient’s life-world and thereby work toward bridging the gap between the patient and the outside world (Gadamer, 1996, p. 163). In discussing the benefits of “the talking cure” Aho and Guigon observe that “the dialogical interplay in which two people engage in bringing to light what is initially inchoate and confused can be seen as a creative act in which new possibilities of understanding and self-formulation are allowed to emerge into the light” (Aho & Guignon, 2011, p. 305). According to Messas et al., a phenomenological approach to therapeutic interview can “help the patient to recalibrate his miscarried position-taking and, finally, to recover his sense of responsibility and agency” (Messas et al., 2018, p.4). Stanghellini proposes that the meaning structures of the patient’s life-world are “rescued” or made explicit by the clinician through hermeneutical investigation (Stanghellini et al., 2019, p. 959). As such, with the space to create suitable hermeneutical resources, the patient carves out alternative meanings of her psychiatric illness experience and can convey these experiences to others; most significantly to the healthcare professionals, who can now offer her the appropriate care.

Bortolan emphasizes that the narrative aspect of phenomenological psychopathology offers the patient not only epistemic insight but is also part of the recovery process. She argues that, by putting one’s phenomenological experience into words, the patient has command over the ambiguous and overwhelming change in her life-world: “This increased sense of control, in turn, inclines us to be more proactive in regulating our feelings, which results in less overwhelming emotions and an increased sense of empowerment” (Bortolan, 2019, p. 1059). Through such speech expression, “certain experiences and behaviors become more understandable and salient” (Bortolan, 2019, p. 1060). In turn, the world for the psychiatric patient begins to appear less alien.

To clarify, it would be overly optimistic to assume that speech expression alone could entirely alleviate the “unworlding” of psychiatric illness. Recall, a drastically altered being-in-the-world is an essential feature of psychiatric illness. Speech expression is just one aspect of the system of interconnected capacities that make up the body schema; reclaiming one aspect of the body schema would not reverse the overall experience of body-world breakdown. Nevertheless, as fundamentally linguistic beings, recovering the subject’s capacity for speech expression is crucial to restoring their being-in-the-world. Speech expression underpins the subject’s relationship with the world as “language is the double of being, and we cannot conceive of an object or idea that comes into the world without words” (Merleau-Ponty, 1976, p.5–6). Thus, recovery of speech expression is necessary (although not sufficient) to restore one’s being-in-the-world. Once the patient’s capacity for speech expression is restored, there is hope of paving the way toward a coherent and more comfortable lived experience, whereby their interactions with objects and others become as inconspicuous and natural as before.

## Conclusion

Through this paper, I have highlighted how hermeneutic strategies in psychiatric healthcare can either ameliorate or exacerbate the experience of “unworlding” for the person with a psychiatric illness. Hermeneutical injustice occurs in psychiatry when the interpretive framework obstructs the patient from understanding their illness-experience because it champions the third-person perspective of the healthcare professional. As such, the patient’s own understanding of their illness is omitted from the collective, clinical understanding of their condition. This is no small loss, as Merleau-Ponty observes that speech expression is a function of the body schema that allows “the human body to celebrate the world and to finally live it” (Merleau-Ponty, 2012, p. 193).

Hermeneutical injustice, in turn, elicits an “unworlding”. The person with psychiatric illness experiences the hermeneutical deficiency as an unexpected absence in their field of experience. Although the intention toward speech expression boils beneath the surface as the person longs to put their experience of psychiatric illness into words, this intention cannot be transformed into action as the necessary hermeneutical resources are missing. Consequently, the person’s movement through the world is stunted due to this gap in one’s system of anticipation. This too has a profound impact on one’s ability to form a social whole with Others, thus further entrenching a gap between the person with psychiatric illness and the world.

On these grounds, I conclude that hermeneutical injustice entrenched in the practices of psychiatric healthcare, contributes to, and perhaps even intensifies, the experience of “unworlding” for the person with psychiatric illness. As such, there is a two-way relationship between unworlding and ineffability in psychiatric illness.

## Notes

1. It is worth stating the distinction between unworlding and “dehumanization” or “depersonalization” whereby one experiences a loss of the unity of the self or a distancing from one’s own body (Sass, 2017, p. 15). While dehumanization and depersonalization may co-exist with unworlding, they are separate experiences.

2. See also Ratclife et al. (2012).

3. “Ready-to-hand” is a term coined by Heidegger to capture the way in which objects in the world offer themselves for practical use, e.g., the cup of tea is “ready-to-hand” as it calls to be drunk.

4. Further discussions on language in pain can be found in Biro (2010).

5. Sass identifies that not all cases of ineffability are a result of “a poverty of speech”. A person with schizophrenia may be willfully silent due to an “indifference to one’s audience” or a desire to keep one’s audience at a distance. It may also be a product of ‘an unfocused or vacillating cognitive style (Sass, 2017, p. 53). Yet “ineffability does seem a particularly central issue, as indicated by how frequently schizophrenic people themselves complain of the inadequacy of language” (ibid).

6. For a detailed account of Merleau-Ponty’s account of intersubjectivity, see Romdenh-Romluc (.^2013^).

7. See Mind (2018).

8. While Perlin popularized the term “sanism”, it was Morton Birnbaum who first coined the term during a trial in 1960 and again in “The Right to Treatment” (1974). A further important contribution to the literature was the term “mentalism”, which captures the same injustice, introduced by Judith Chamberlain in 1975.

9. I do not suggest that healthcare professionals intentionally downgrade the credibility of their patients. When hermeneutical injustice occurs, it is through an implicit structural prejudice within the healthcare system itself despite the healthcare professional’s best efforts to help their patient. Indeed, as Foucault suggests, the source is a historically contingent social-epistemic structure: “the underlying structures that form the context for their thinking” (Gutting, 2005, p. 32).

10. This is referred to as “expressive restrictions” in the healthcare context by Kidd and Carel (2018).

11. For further work on hermeneutical injustice in psychiatry see Spencer and Carel (2021), Ritunnano (2022), Kidd et al. (2022).

12. Lisa Guenther (2017) made a similar observation. Guenther aims to enrich Fricker’s account of “hermeneutical sensibility” with Merleau-Ponty’s account of Self and Other. I critique this argument in my doctoral thesis (Spencer, 2021).
